# Evaluation of the reasons for failure in teeth with vital amputation treatment

**DOI:** 10.1186/s12903-023-03171-z

**Published:** 2023-07-06

**Authors:** Semsettin Yildiz, M. Sinan Dogan, Mehmet Emin Dogan

**Affiliations:** 1 Department of Pediatric Dentistry, Faculty of Dentistry, Fırat University, Elazıg, Turkey; 2 Department of Pediatric Dentistry, Faculty of Dentistry, Harran University, Sanlıurfa, Turkey; 3 Department of Dentomaxillofacial Radiology, Faculty of Dentistry, Harran University, Sanlıurfa, Turkey

**Keywords:** Deciduous tooth, Vital amputation, Calcium hydroxide

## Abstract

**Aim:**

This study aims to evaluate the primary teeth undergoing amputation due to dental caries or trauma clinically and radiologically.

**Material and methods:**

The amputation treatment of 90 primary teeth of 58 patients (Female: 20, Male: 38) aged 4–11 years was evaluated clinically and radiologically. Calcium Hydroxide was used for amputation in this study. Composite or amalgam was preferred as filling material in the same session of the patients. Clinical/radiological (Periapical/Panoramic X-ray) examination was performed on the teeth that were unsuccessful in treatment, on the day of the patient's complaint, and at the end of 1 year in the others.

**Results:**

According to the clinical and radiological findings of the patients, 14.4% of the boys and 12.3% of the girls were unsuccessful. Amputation in male was a need in the 6–7 age group with a rate of 44.6% at most. Amputation in females was a need in the 8–9 age group with a rate of 52% at most.

**Conclusion:**

Success in amputation treatment depends on the tooth, the dentist, and the dental material applied.

## Introduction

Despite the decreasing prevalence of dental caries today, this disease continues to be one of the critical public health problems affecting children and adults [1]. Tooth decay is a significant health problem that results in pain and reduces the quality of life of children. However, painless and untreated decayed teeth can cause severe dental and systemic problems [2].

The primary purpose of dental caries management is to preserve the tooth's vitality and prevent tooth hard tissue loss. However, due to the anatomical structure of primary teeth, low mineralization, and high-risk factors, dental caries progresses rapidly [3].

Due to the rapid progression of dental caries in children and a delayed visit to the dentist due to socioeconomic reasons, dental caries affect the pulp. Depending on the size of dental caries and their effect on the pulp, pulp coating (direct or indirect), pulpotomy and pulpectomy are the treatment options [4].

Pulpotomy is a procedure to remove the coronal pulp affected by dental caries or trauma and preserve the vitality of the root pulp. For this purpose, different techniques and materials such as ferric sulfate, glutaraldehyde, Mineral trioxide aggregate (MTA), calcium hydroxide, electrosurgery, and laser therapy are used [5].

Microleakage-free restorations and cavity closure materials are essential for successful treatment in pulpotomy treatment. Because one of the most common causes of failure is the entry of bacteria into the pulp from the salivary environment through the open dentinal tubules. Preventing marginal leakage is essential to vital pulp therapy, as bacterial contamination and infection are significant threats to pulp healing [6]. After pulpotomy, amalgam, glass ionomer, composite, and stainless steel crowns can be used [6, 7]. The restoration to be made is determined according to the amount of tooth tissue remaining after the tooth decay is removed and the time of the tooth falling out [8].

There is a study in the literature evaluating vital pulpotomy failure [9]. However, the filling material used was not evaluated. In this study, the effect of the filling material used after vital pulpotomy on the success of the treatment was investigated.

The aim of this study was to evaluate the clinical and radiological success of primary molar amputations with calcium hydroxide according to different restorative materials.

The null hypothesis (H_0_) there is no difference in the success rate of the restorative materials used after amputation.

## Material and method

Amputation was performed on 90 teeth of 58 patients aged 4–11. G-power version 3.1.9.7 was used for power analysis and the sample size was found to be 78 at the 5% error interval and 90% confidence interval. In this study, 90 teeth were included.

The criteria for teeth inclusion into the study were as follows;

No clinical or radiographic pathological findings; the pulp is opened during caries removal; Patients with bleeding control for 5 min after coronal pulp amputation, teeth within normal limits, and patients without any systemic disease were included in the study. Exclusion criteria; patients with systemic disease, syndrome, noncooperative, patient with lesion cyst, tumor etc. were not included in the study.

In the clinical examination performed at the control appointment, Sensitivity to percussion and palpation, spontaneous or causative pain, gingival discoloration, fistula or abscess, pathological mobility were evaluated.

Radiolucency in the periapical or furcation region, internal and external pathological root resorption, and expansion of the periodontal ligament were evaluated in the radiological examination. Teeth with any of these clinical and radiological findings were considered to be unsuccessful.

All carious tissues were removed under local anesthesia. Coronal pulp tissue was removed using a conventional technique. Bleeding was controlled with moist cotton pellets. A sterile powder of calcium hydroxide mixed with distilled water was applied to the root pulp and gently adapted with a clean cotton pellet to perform calcium hydroxide amputation. After using glass ionomer cement (Fuji Lining LC, GC, Japan) as a base, final restorations were made with amalgam or composite filling. Clinical and radiological follow-up was 12 months.

This study was conducted with the approval of Harran University Clinical Research Ethics Committee dated 21.06.2021 and numbered HRU 21.12.29.

### Statistical analysis

SPSS (IBM Corp, Armonk, NY) statistics 25 package program was used in the analysis of the data. In order to control the normal distribution of the obtained data, evaluation was made with the Shapiro–Wilk test. Number (N), percentage (%), minimum and maximum values were analyzed with descriptive statistics. The Mann–Whitney U test was used to evaluate the differences between the groups. Pearson chi-square analysis was used to examine the relationships between categorical variables. Significance level was accepted as *p* < 0.05.

## Results

In this study consisted of 38 (65.5%) males, 20 (34.5%) females, mean age 7 ± 1.8 years, between 4–11 years of age. The mean age of males was 6 ± 1.4, and the mean age of females was 8 ± 2.1. According to the clinical and radiological findings of the patients, the success rate was 73.3%, the failure 26.7%. Failure was found in 14.4% of male and 12.3% of female. Amputation in male was a need in the 6–7 age group with a rate of 44.6% at most (Table 1). In males, 18.2% failure was observed in composite teeth and 20.4% in amalgam. Amputation in females was a need in the 8–9 age group with a rate of 52% at most (Table 2). No significant relationship was observed between age groups in terms of achievement status (*p* > 0.05) (Tables 3 and 4). While no loss was observed in composite teeth in girls, 52.4% failure was observed in amalgam-applied teeth. The number of decayed teeth was mostly detected in the 6–7 age group with a rate of 44.8% in males. Considering the achievement status by gender, it was found to be significantly higher in males (*p* < 0.05) (Table 5). While the success rate was 57.8% for males, it was 15.6% for females. Considering the success rate according to the material, the success of amalgam was found to be significantly higher in males than in females (*p* = 0.006). While the success rate of amalgam in males was found 79.6%, it was 47.6% in females.

**Table 1:** Distribution of decayed teeth, number of amputated teeth and internal resorption in males by age groups

Male	5 years and under	6–7 years	8–9 years	10 years and older	**Total**
	N (%)	N (%)	N (%)	N (%)	N (%)
**Number of patients**	13 (34.2)	16 (42.1)	7 (18.5)	2 (5.2)	38 (100)
**Number of amputated teeth**	21 (32.4)	29 (44.6)	12 (18.4)	3 (4.6)	65 (100)
**Number of decayed teeth**	116 (35.5)	146 (44.8)	48 (14.7)	16 (5.0)	326 (100)
**Failed Restoration**	1	0	2	0	3
**Internal resorption**	2	6	0	0	8

*N* number, % percent

**Table 2:** Distribution of decayed teeth, number of amputated teeth and internal resorption in females by age groups

Female	5 years and under	6–7 years	8–9 years	10 years and older	**Total**
	N (%)	N (%)	N (%)	N (%)	N (%)
**Number of patients**	4 ( 20)	3 (15)	10 (50)	3 (15)	20 (100)
**A number of teeth were amputated**	4 (16)	4 (16)	13 (52)	4 (16)	25 (100)
**Number of decayed teeth**	33 (21)	31 (20)	77 (49)	16 (10)	157 (100)
**Failed restoration**	1	0	0	0	1
**Internal resorption**	1	0	1	0	2

**Table 3:** Distribution of the success status of the restoration material according to age groups in men

Male	**Restoration material**	**Composite**	**Amalgam**	**Others**	**Total**	***P*** **-value**
5 years and under	Successful	4	14	0	18	0.676
	Unsuccessful	1	2	0	3	
6–7 Years	Successful	3	19	0	22	0.302
	Unsuccessful	0	7	0	7	
8–9 years	Successful	2	7	0	9	0.700
	Unsuccessful	1	2	0	3	
10 years and older	Successful	0	3	0	3	-
	Unsuccessful	0	0	0	0

**Table 4:** Distribution of the success status of the restoration material according to age groups in female

Female	**Restoration material**	**Composite**	**Amalgam**	**Others**	**Total**	***P*** **-value**
5 years and under	Successful	1	1	0	2	0.248
	Unsuccessful	0	2	0	2	
6–7 years	Successful	3	1	0	4	-
	Unsuccessful	0	0	0	0	
8–9 years	Successful	0	5	0	5	-
	Unsuccessful	0	8	0	8	
10 years and older	Successful	0	3	0	3	-
	Unsuccessful	0	1	0	1

**Table 5:** Distribution of the success status of the restoration material by general gender

	Male	Female	*P*-value
	N (%)	N (%)	
COMPOSİTE			
Successful	9 (81.8)	4 (100)	0.360
Unsuccessful	2 (18.2)	0 (0)	
Total	11( 100)	4 (100)	
AMALGAM			
Successful	43 (79.6)	10 (47.6)	0.006*
Unsuccessful	11 (20.4)	11 (52.4)	
Total	54 (100)	21 (100)	

^*^ *p* < 0.05

## Discussion

Amputation is a solid essential determinant of one or more clinical and radiological findings such as pain, inflammation, radiolucency areas, and internal or external root resorptions. Several factors affect the success of amputation treatment in primary molar teeth, such as the inability to diagnose chronic periapical infection, the irritant effect of the amputation material on the pulp tissue, and microleakage [5, 9–11]. Kulkarni et al. the causes of failure due to superstructure restorations used in deciduous teeth that underwent amputation treatment; The coronal pulp is not entirely cut and removed, the cavity is not thoroughly cleaned of caries, and secondary caries occurs, the filling material used is overflowing or incompletely placed, the edge adaptation of the restorative filling material is not complete, especially in the neck area, the stainless steel crown is not well adapted to the sleeve, the stainless steel crown is used for bonding. They stated that the cement is undergoing resorption [2]. As a result of our study, a failure (Unsuccessful) rate of 10,86% was detected due to failed restoration and internal resorption.

Researchers have agreed that the ideal pulp treatment is to keep the root pulp vital, healthy, and entirely covered by the odontoblast layer. Calcium hydroxide (Ca (OH)_2_) is the first amputation agent that has been shown to have the capacity to stimulate dentin regeneration and to keep the root pulp alive. The material is a substance that has been widely used in dentistry for many years due to its antibacterial properties, and its biocompatibility is compared with other antibacterial agents [12].

Ca (OH)_2_ is still the most classical agent that can stimulate the regeneration of dentin and maintains the vitality of the root pulp [13]. As a deciduous tooth amputation material, it is cheap, easy to use, and practical. It is aimed to repeatedly question the reasons for the failure of Ca (OH)_2_ and to eliminate these negative aspects [14]. In our study, it was found that the failure rate in primary molar tooth amputations performed with calcium hydroxide increased with age. We think this result is related to the aging of the primary tooth pulp.

Percinoto et al.; In their studies in which the efficacy of Ca (OH)_2_ and Mineral trioxide aggregate (MTA) amputations were compared radiographically, there was no statistical difference at the end of the 12^th^ month, but 13.33% for the Ca (OH)_2_ group; They reported a 4.44% overall failure for the MTA group. Researchers have stated that both materials can succeed in amputation treatments with careful case selection. In this study, the limited follow-up period of 12 months and the temporary placement of cotton pellets impregnated with a corticosteroid antibiotic solution into the pulp cavity to prevent pulp irritation, prevent inflammation, and increase repair capacity after bleeding control may have contained the success rates of Ca(OH)_2_ [15].

Sonmez et al. if; At the end of 24 months, the overall success rate was 76.9% for Formocresol; 73.3% for ferric sulfate; It was found to be 46.1% for Ca(OH)_2,_ and 66.6% for MTA. Although there was no significant relationship between the groups at the end of the study, it was stated that the Ca(OH)_2_ group was relatively unsuccessful compared to the other three groups [16]. Moretti et al. At the end of the 24-month follow-up, it was observed that the MTA group had 100% radiographic success. They reported that 35.7% failure due to internal resorption was observed in the group treated with Ca(OH)_2_ in the 3rd month. The overall failure rate increased to 64% at the end of the follow-up period, and this difference was statistically significant. In their study, in which the researchers stated the importance of bleeding control, the same problem was valid for both Ca(OH)_2_ and MTA; however, due to the inability to prevent clot formation in Ca(OH)_2_ amputations, internal resorption may have been observed at a high rate due to the failure to provide contact between the material and the pulp; They concluded that these results might have been obtained because MTA can harden even in the presence of moisture compared to Ca(OH)_2_ and its superior hiding power [17].

Starting from the 6th month, the Ca(OH)_2_ paste starts to soften and loses its integrity. As a result of the formation of cavities under the restoration, it can create a passageway for bacterial infection. It has been reported in previous studies that failure in Ca(OH)_2_ amputations is generally seen from the 6th month [18, 19].

In this study, parallel to the literature studies, we think this failure is due to the internal resorption of Ca(OH)_2_ and the resolution of Ca(OH)_2_ over time.

Some researchers do not see internal resorption as a failure [20–22]. He explains internal resorption as a necrotic tissue formation due to clinical pulpitis [17, 23]. A failure can be mentioned when this pathology includes permanent tooth germ and a defect after the eruption of enamel causes it [24]. Today, this is seen as a radiological failure and does not need to be treated immediately. It is recommended to follow. This idea was adopted because it showed no signs of clinical failure. In addition, every deciduous tooth may not be intervened in every pathological finding because the presence of primary teeth may not affect the permanent tooth germ. It needs to be followed. It is recommended to shoot when it shows symptoms. Regardless of the material used, it was emphasized that follow-up is important [17]. We used radiographic data and emerging symptoms (pain, swelling) as criteria for failure in the study.

Healing of the washer after amputation treatment is that this medicament, rather than the material used, should cover the pulp well, create a good biological barrier, and the restoration should be done in a way that prevents microleakage in the long term [15, 25, 26]. Prefabricated steel crowns (PPC) are recommended for vitally amputated teeth [12, 24, 25, 27–30].

## Conclusion

Amalgam filling was found to be more successful in males than females.

- The need for amputation was needed at most around 6–7 years of age.
- It has been seen that the need for amputation treatment is more in male. There was no significant relationship in age groups between the number of decayed teeth and the number of amputated teeth.
- In fillings made after amputation, the success rate of composite is lower than amalgam. There was no significant relationship between the filling material and the success status when looking at the age groups.
- The failure rate in primary molar amputations performed with calcium hydroxide has been observed to increase with age, and we think that this is related to the aging of the primary tooth pulp.
- We concluded that failure is due to the internal resorption of Ca(OH)_2_ and the resolution of Ca(OH)_2_ over time.
- As a result, we feel this article may be useful for practitioners and researchers, although it is apparent there continues to be a need for increased research.

## Data Availability

The datasets used and analyzed during the current study are available from the corresponding author upon reasonable request.
